# Treatment of Residual, Recurrent, or Metastatic Intracranial Hemangiopericytomas With Stereotactic Radiotherapy Using CyberKnife

**DOI:** 10.3389/fonc.2021.577054

**Published:** 2021-03-03

**Authors:** Lichao Huang, Jingmin Bai, Yanyang Zhang, Zhiqiang Cui, Zhizhong Zhang, Jiwei Li, Jinyuan Wang, Xinguang Yu, Zhipei Ling, Baolin Qu, Longsheng Pan

**Affiliations:** ^1^ Department of Neurosurgery, The First Medical Center of PLA General Hospital, Beijing, China; ^2^ Department of Neurosurgery, The Hospital of 81st Group Army PLA, Zhangjiakou, China; ^3^ Department of Radiation Oncology, The First Medical Center of PLA General Hospital, Beijing, China

**Keywords:** stereotactic radiotherapy, CyberKnife, hemangiopericytomas, tumor control, management

## Abstract

**Purpose:**

Hemangiopericytomas are aggressive tumors known for their recurrence. The purpose of this study was to evaluate the management of residual, recurrent, and metastatic intracranial hemangiopericytomas using CyberKnife (CK) stereotactic radiotherapy (SRT).

**Materials and Methods:**

Data were collected from 15 patients (28 tumors; eight men and seven women; 32–58 years) with residual, recurrent, or metastatic intracranial hemangiopericytomas, who were treated with stereotactic radiotherapy using CyberKnife between January 2014 and August 2019. All patients had previously been treated with surgical resection. Initial tumor volumes ranged from 0.84 to 67.2 cm^3^, with a mean volume of 13.06 cm^3^. The mean marginal and maximum radiosurgical doses to the tumors were 21.1 and 28.76 Gy, respectively. The mean follow-up time for tumors was 34.5 months, ranging from 13 to 77 months.

**Results:**

15 patients were alive after treatment; the mean post-diagnosis survival at censoring was 45.6 months (range 13–77 months). The volumes of the 28 tumors in the 15 followed patients were calculated after treatment. Postoperative magnetic resonance imaging revealed a mean tumor volume of 6.72 cm^3^ and a range of 0–67.2 cm^3^, with the volumes being significantly lower than pretreatment values. Follow-up imaging studies demonstrated tumor disappearance in seven (25%) of 28 tumors, reduction in 14 (50%), stability in one (3.57%), and recurrence in six (21.4%). Total tumor control was achieved in 22 (78.5%) of 28 tumors. The tumor grade and fraction time were not significantly associated with progression-free survival. Intracranial metastasis occurred in three patients, and extraneural metastasis in one patient.

**Conclusions:**

On the basis of the current results, stereotactic radiotherapy using CyberKnife is an effective and safe option for residual, recurrent, and metastatic intracranial hemangiopericytomas. Long-term close clinical and imaging follow-up is also necessary.

## Introduction

Hemangiopericytomas (HPCs) are rare tumors that exhibit a high incidence of local recurrence and distant metastasis, even after gross-total resection (1). Central nervous systems HPCs are uncommon, accounting for only 0.4% of all primary intracranial tumors and 2.4% of meningeal tumors (2). Central nervous system (CNS) HPC usually occurs in adults with an average diagnostic age between 40 and 50 years (3). The World Health Organization(WHO) guidelines combine solitary fibrous tumors and HPC into the single entry solitary fibrous tumor(SFT)/HPC, which was classified into three variants in 2016: grades I, II, and III (4). An analysis of surveillance, epidemiology, and end results in 655 patients and a review of CNS (199) and extra-CNS HPCs (456) showed 5- and 10-year overall survival rates of 80 and 54%, respectively. Patients with extracranial HPCs had worse outcomes, with 5- and 10-year overall survival rates of 58 and 44%, respectively (3).

HPCs frequently show involvement of adjacent dural sinuses and the skull base, which can make gross-total resection a challenging, and at times, unrealistic goal. In almost all cases, the initial treatment of larger intracranial HPCs is resection, with surgical excision remaining the gold standard treatment. The local control, progression-free survival, and overall survival rates of patients receiving radiotherapy seem to be higher than those who do not receive radiotherapy (5–7), but the efficacy of adjuvant radiotherapy is still under study. The optimal management of recurrent or residual intracranial HPCs presents a challenge. The roles of Gamma Knife Radiosurgery (GKRS) and CK SRT in the treatment of HPC have been previously described, with tumor control rates ranging from 46 to 100% (2). However, the published reports describing the use of CK SRT in the treatment of HPCs are limited. Furthermore, the optimal dose for successful local control of HPCs without adverse effects remains unclear. In this study, we evaluated the safety and efficacy of SRT using the CK system in 15 patients with residual, recurrent, and metastatic intracranial HPCs.

## Materials and Methods

### Patient Population

This study enrolled 15 patients with HPCs who were treated in our institute between January 2014 and August 2019. Written informed consent was obtained from each participant prior to study inclusion, and the study was approved by the local ethics committee of our institute (No. S2018-119-01). The patient inclusion criteria were: (1) all patients received craniotomy and had histopathologically confirmed diagnoses; (2) all patients received magnetic resonance imaging (MRI), including T1-weighted, T2-weighted, contrast-enhanced T1-weighted and FLAIR sequences; (3) all HPCs were documented as residual, metastatic, or recurrent lesions. Clinical data including sex, age, diagnosis, baseline neurological symptoms, and lesion location were collected. In addition, information on the prescription dose, planning target volume, and concurrent therapy was also obtained.

### CK Technique

Before treatment, all patients underwent planning CT (Siemens, Forchheim, Germany) and 3.0T MRI (Siemens, Erlangan, Germany) with slice thickness of 1 mm. Rigid fusion registration was performed between the MRI T1-weighted contrast-enhance sequences and CT scans using MIM Maestro 6.5.4 image processing software (MIM Software Inc., Cleveland, Ohio, USA). The gross tumor volume (GTV) was determined from the fused image, and the planning target volume (PTV) was defined a region extending 1.5 mm outside the gross tumor volume. The treatment plans varied according to the size of the treated tumor, its location in relation to critical structures, and the history of prior radiation. All 28 tumors in the 15 patients were treated with SRT using CK system (Accuray Inc., Sunnyvale, CA).

### Follow-Up Evaluation

All patients were interviewed and clinically evaluated to update their clinical and personal data. Follow-up brain MRI was acquired from all 15 patients 3 months after CK, and then regularly at 3- to 6-month intervals. At each follow-up, tumor volumes were determined from the brain MRI using the coniglobus formula: V = 1/6π × a (diameter length) × b (diameter width) × m (slice thickness) × c (slice number). The follow-up time ranged from 13 to 77 months, with a mean of 34.5 months. The tumor volume response was classified in the following manner: ‘disappeared’ (100% decrease in tumor volume), ‘reduction’ (25–99% decrease in tumor volume), ‘stable’ (≤25% decrease or 25% increase in tumor volume), and ‘recurrence’ (>25% increase).

### Statistical Analysis

Statistical analysis was performed using SPSS version 22.0 (SPSS Inc., Chicago, IL, USA). Overall survival and progression-free survival were calculated using Kaplan–Meier plots. Univariate analysis was performed on the Kaplan–Meier curves using the log-rank test. Statistical significance was set at p < 0.05.

## Results

### Imaging Outcomes

The 15 patients consisted of eight men (53.4%) and seven women (46.6%), with a median age of 43 years (range 32–58 years) at the time of initial CK therapy (**Table 1**). All patients were previously treated with surgical resection and had histopathologically confirmed diagnoses. Eight patients had a WHO classification of grade II, two of grades II–III, and five of grade III. One patient had undergone four craniotomies before CK, two patients had undergone three, two patients had undergone two, and 10 patients had undergone one. Four patients had undergone GK treatment after surgical resection, and all had infield recurrence. The mean time from surgery to CK treatment was 14.5 months (range 1–96 months). The patients’ main symptoms included headache (n = 6), visual impairment (n = 2), and hemiplegia and alalia (n = 1), with six being asymptomatic (**Table 1**). All intracranial HPCs were documented as residual (8), recurrent (9), and/or metastatic (4). Two patients underwent five CK SRT treatments, one patient underwent four treatments, four patients underwent two treatments, and eight patients underwent one treatment. The most recent CK treatments in patients 1, 3, 8 were covered by a follow-up period of less than 12 months at the time of censoring and are not included in the statistical analysis. For the total of 28 tumors in 15 patients, the mean tumor volume was 13.06 cm^3^ (range 0.84–67.2 cm^3^). The 28 tumors were located in a myriad of locations, including the temporal lobe (n = 2), anterior skull base (n = 3), fourth ventricle (n = 1), parietal and parasagittal region (n = 5), tentorium cerebelli (n = 10), cerebellum(n = 2), confluence of the sinuses (n = 2), cavernous sinus (n = 1), sellar region (n = 1), and orbital apex (n = 1, **Supplementary Table 2**). Among the 28 tumors, four tumors were treated twice and were regarded as recurrent. One patient accepted anti-angiogenesis therapies after metastasis, according to the professional advice of an oncologist.

**Table 1:** Patient characteristics.

Number of patients	Gender	Age at onset(years)	Clinical presentation	Number of craniotomy before CK	Radiation therapy before CK(time, dose)	Grade	Time to CK post-surgery (month)	Number of CK treatments	Site
1	F	58	Headache, walking unsteadily	3	GKRS (1, 15Gy)	III	96	5	Left anterior skull base, temporal lobe, and the fourth ventricle
2	M	49	Headache	1	None	II	1	1	Left parietal and parasagittal
3	M	38	Asymptomatic	1	None	II–III	5	5	Tentorium cerebelli, confluence of sinuses
4	M	38	Headache	1	None	III	8	4	Tentorium cerebelli
5	M	32	Visual impairment	2	GKRS (1, 14.5Gy)	III	2	2	Cavernous sinus, orbital apex
6	F	37	Headache	3	None	II	2	2	Anterior skull base
7	M	43	Asymptomatic	4	GKRS (2, 15Gy, 13Gy)	III	1	1	Right cerebellum
8	F	49	Asymptomatic	2	GKRS (2, 15Gy, 15Gy)	II	19	1	Right parietal and parasagittal
9	M	48	Hemiplegia, alalia	1	None	II	41	1	Right tentorium cerebelli
10	F	46	Asymptomatic	1	None	III	1	1	Left parietal
11	M	34	Headache	1	None	II	1	2	Confluence of sinuses
12	F	42	Headache	1	None	II	2	1	Right parietal
13	F	46	Asymptomatic	1	None	II	36	1	Left cerebellum
14	M	42	Visual impairment	1	None	II	1	1	Sellar region
15	F	46	Asymptomatic	1	None	II–III	2	1	Right parietal

M, male; F, female; CK, CyberKnife; GKRS, Gamma Knife Radiosurgery.

Twenty-four tumors required treatment in three fractions, one tumor required two fractions, and three tumors required a single session. The mean marginal dose to the tumors was 21.1 Gy (range 14–27 Gy) for the initial CK SRT, and the mean maximum, mean, and minimum radiosurgical doses were 28.86 Gy (range 20–33.75 Gy), 25.53 Gy (range 17.08–30.83 Gy) and 15.74 Gy (range 4.02–26.52 Gy), respectively. The mean isodose line was 73.03% (range 70–80%, **Supplementary Table 2**). In the three fraction treatments, the mean marginal dose was 21.65 Gy (range 18–27 Gy), and the mean maximum, mean, and minimum radiosurgical doses were 29.39 Gy (25.71–33.75 Gy), 25.06 Gy (21.87–30.83 Gy), and 15.72 Gy (4.02–26.52 Gy), respectively. In the two fraction treatment, the marginal dose was 22 Gy, and the maximum, mean, and minimum radiosurgical doses were 31.42, 28.2, and 20.49 Gy, respectively. In the process of one fraction treatments, the mean marginal dose was 16.6 Gy(range 14–20 Gy), the maximum, mean, and minimum radiosurgical doses were 23.8 Gy (20–28.57 Gy), 20.3 Gy (17.08–24.55 Gy), and 14.3 Gy (11.54–19.63 Gy).

**Figure 1:**
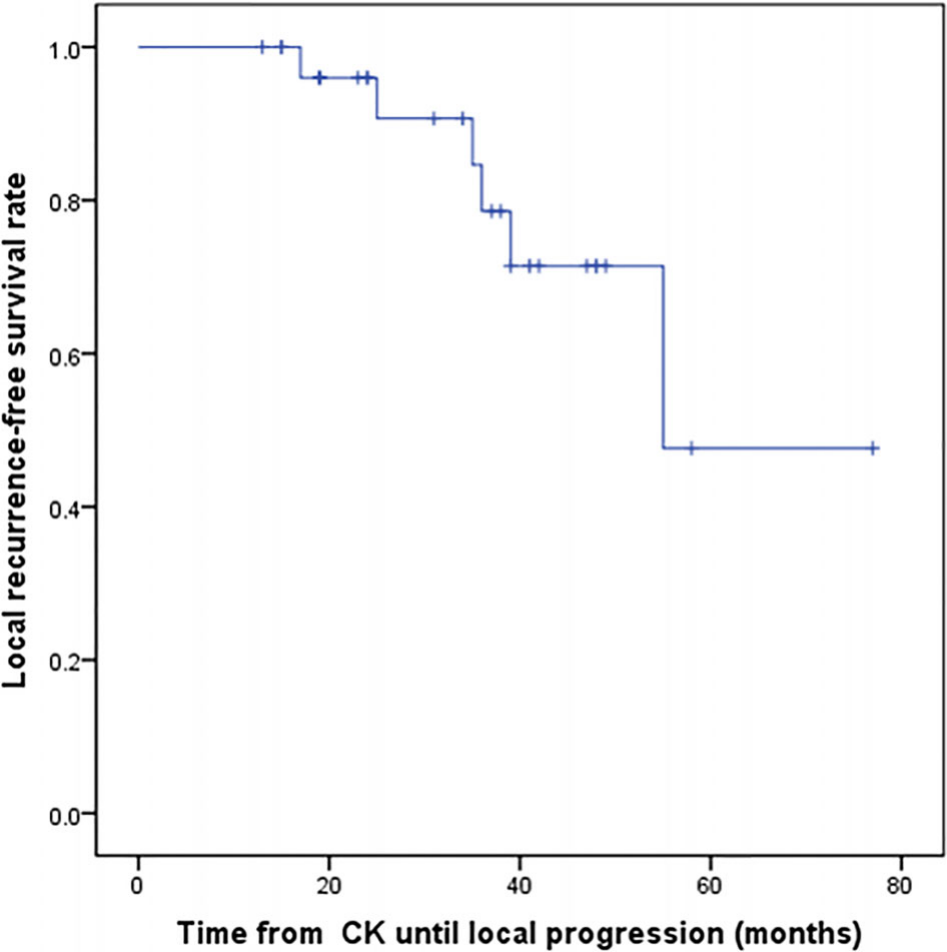
Kaplan–Meier progression-free survival curves after CK treatment for all patients.

**Figure 2:**
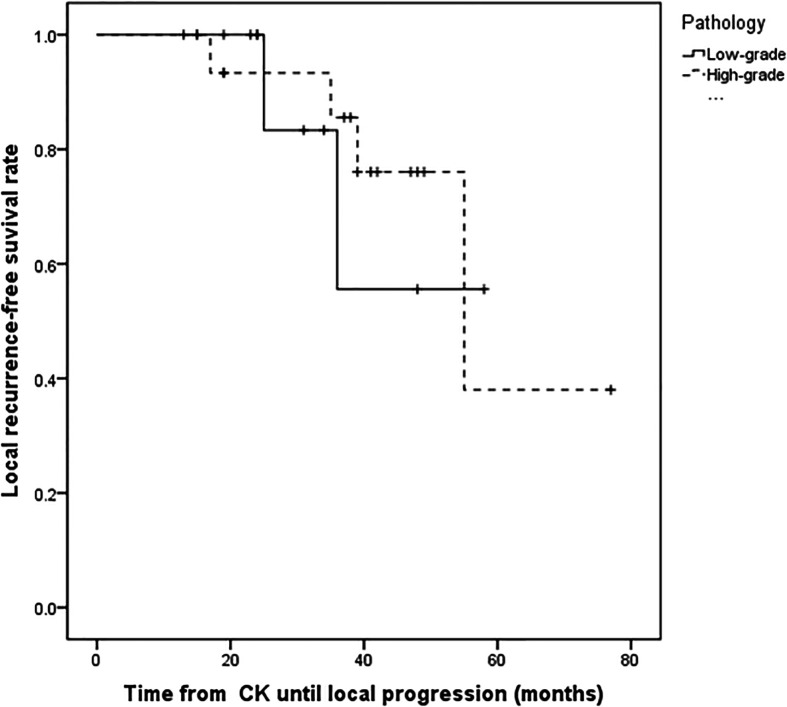
Kaplan–Meier progression-free survival curves for different pathology. There was no statistically significant difference between patients with high-grade tumors and low-grade tumors (P = 0.754).

**Figure 3:**
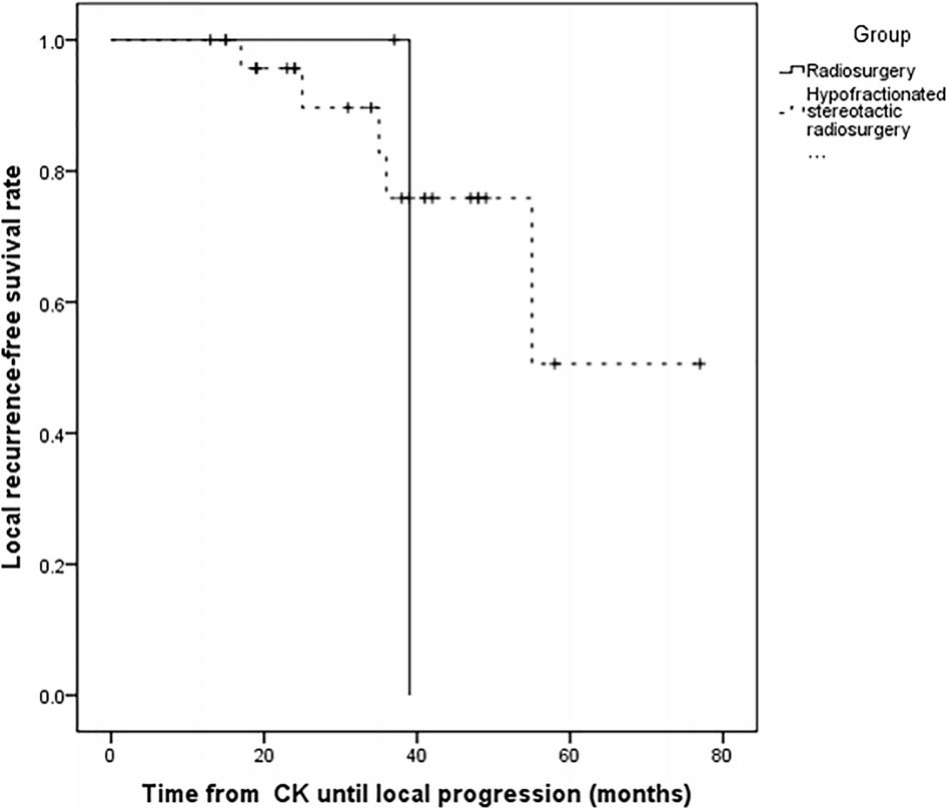
Kaplan–Meier progression-free survival curves for different fraction time. There was no statistically significant difference between radiosurgery and hypofractionated stereotactic radiosurgery (P = 0.529).

**Figure 4:**
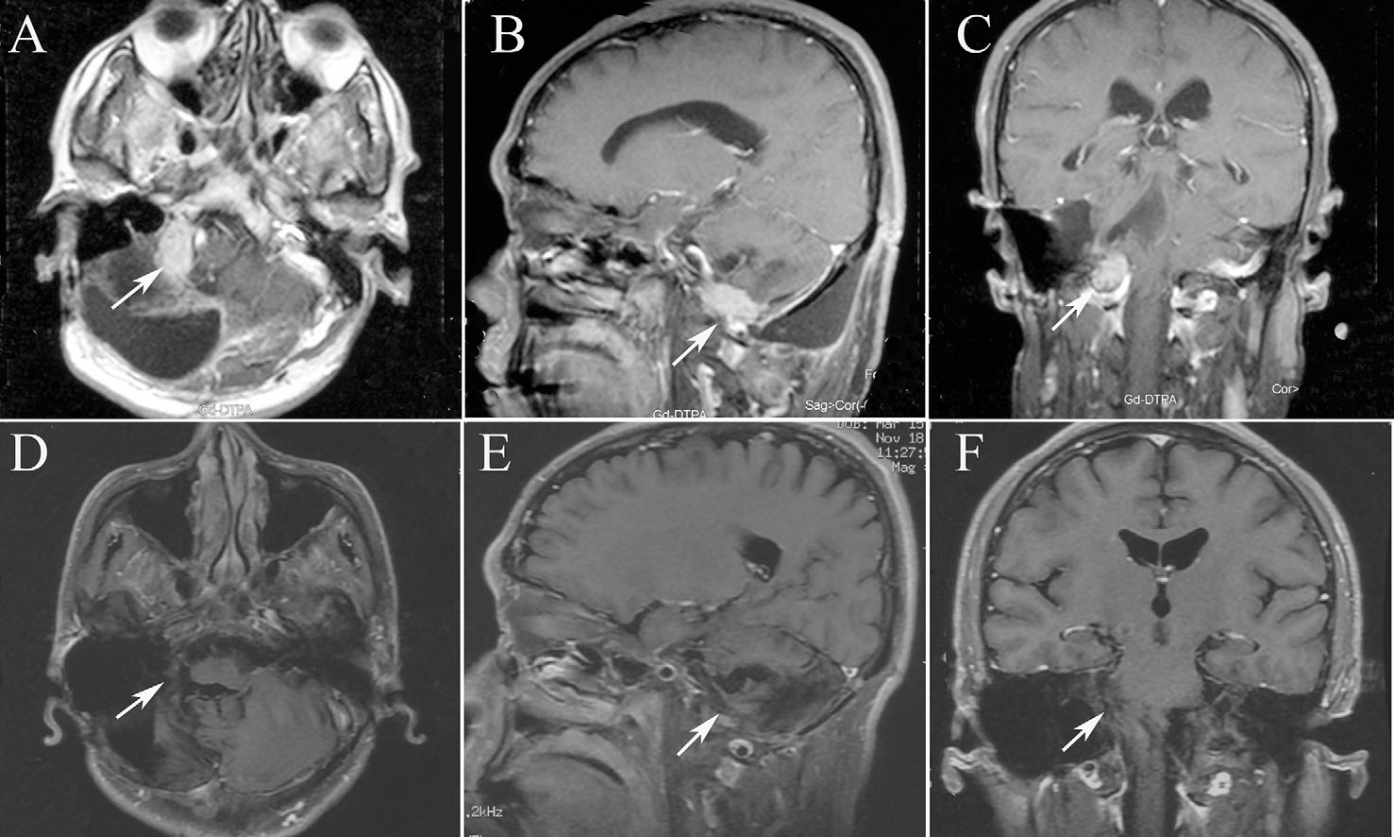
The MRI images of patient 7, tumor 16. **(A)** Axial T1-weighted contrast-enhanced image showing residual enhancement and a pretreatment tumor volume of 11.33 cm^3^ (tumor 16); **(B)** Sagittal T1-weighted contrast-enhanced image; **(C)** Coronal T1-weighted contrast-enhanced image; **(D)** Axial T1-weighted contrast-enhanced image after 13 months, the tumor disappeared. **(E)** Sagittal T1-weighted contrast-enhanced image after 13 months; **(F)** Coronal T1-weighted contrast-enhanced image after 13 months.

**Figure 5:**
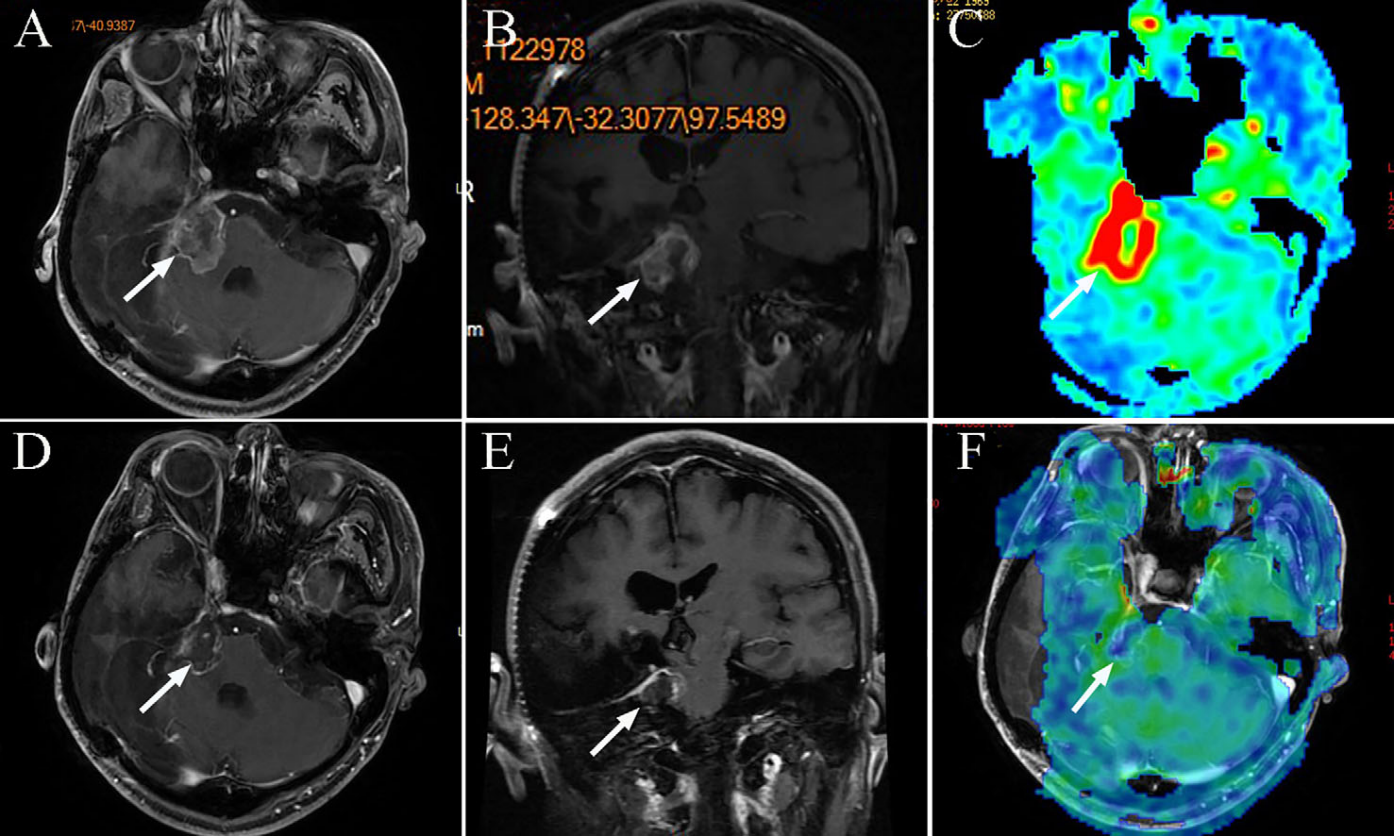
The MRI images of patient 9, tumor 20. **(A)** Axial T1-weighted contrast-enhanced image showing residual enhancement and a pretreatment tumor volume of 13.41 cm^3^ (tumor 20); **(B)** Coronal T1-weighted contrast-enhanced image; **(C)** Axial three dimensional arterial spin labeling (3D-ASL) image showing high perfusion; **(D)** Axial T1-weighted contrast-enhanced image after 12 months, the tumor reduced. **(E)** Coronal T1-weighted contrast-enhanced image after 12 months; **(F)** Axial 3DASL image showing low perfusion after 12 months.

For the all surviving 15 patients, the mean post-diagnosis survival at censoring was 45.6 months (range 13–77). But the number of events required for the survival analysis has not been reached, so no formal statistical comparison has been performed. The follow-up time ranged from 13 to 77 months, with a mean of 34.5 months. Postoperative MRI revealed tumor volumes ranging from 0 to 67.2 cm^3^, with a mean value of 6.72 cm^3^, volumes that were significantly lower than on pretreatment MRI. The follow-up imaging studies demonstrated that seven of 28 (25%) tumors had disappeared at a mean of 30.14 months (range 3–48 months) after CK, 14 (50%) had reduced, one (3.57%) was stable, and six (21.4%) had recurred after reduction at mean of 33.1 months (range 15–55 months) after CK therapy. Tumor recurrence was founded at a time interval of 3 or 6 months following the reduction. Total tumor control was achieved in 22 (78.5%) of 28 tumors, with the actuarial local control rate of 28 tumors at 1-year being 100% (**Figure 1**). No correlation between treatment dose, tumor volume, and tumor response was apparent in these patients. Total tumor control was achieved in 13 of 17 (76.4%) tumors in the high-grade group and nine of 11 (81.8%) tumors in the low-grade group. The progression-free survival curves in **Figure 2** no statistically significant difference between patients with high-grade tumors and low-grade tumors (p = 0.754). In the radiosurgery and hypofractionated groups, total tumor control was achieved in two of three (75%) tumors, and 20 of 25 tumors (80%), respectively. The progression-free survival curves showed no statistically significant differences between radiosurgery and hypofractionated stereotactic radiosurgery (p = 0.529; **Figure 3**). Intracranial metastasis occurred in three patients and extraneural metastasis in one patient. Six tumors (one low-grade tumor and five high-grade tumors) showed recurrence after undergoing a reduction in volume. No complications occurred after treatment in any patient in this series. **Figures 4** and **5** show tumor numbers 18 and 20 before and after CK therapy. **Supplementary Table 2** summarizes the patient characteristics and radiotherapy parameters of the treatment plans.

#### Clinical Outcomes

Clinical symptoms were followed in all 15 patients. Of those with adequate follow up data, six patients reported resolution of headaches, and eight indicated no change in symptoms, while no patients described worsening of the initial clinical presentation. All cranial nerve deficits present at initial presentation remained, with no improvement or worsening.

## Discussion

HPC’s are derived from fibro-histiocytic precursor cells, the pericytes of Zimmerman (10). HPCs resemble meningiomas both clinically and radiographically, but are known for their aggressiveness, high recurrence rates, and propensity for extracranial metastasis (11). Therefore, HPCs differ from meningiomas and require systemic management and long-term follow-up. The efficacy of radiotherapy and chemotherapy for recurrent intracranial HPCs remains unclear because of the scarcity and deficiencies of the available clinical data (8, 12). The optimal CK SRT dose for successful local control of HPCs without adverse effects also remains unclear.

Resection provides the benefits of histological confirmation and reduces mass effects, and is usually the primary treatment for HPC. In previous studies, gross-total resection (GTR) was significantly associated with longer progression-free survival and overall survival. In a systematic review of 523 patients with CNS HPCs (13), the mean survival in patients undergoing surgery with complete resection was 157.97 months. In contrast, patients with an incomplete resection had a mean survival of 110.75 months, which was observed to be related to a higher incidence of mortality. Although gross-total resection can be challenging as the tumor frequently infiltrates the sinuses and is highly vascular, it should still be the primary treatment goal in patients with HPC. Patients with tumors of the posterior fossa had a shorter survival time (median 10.75 *vs.* 15.6 years) than those with tumors located elsewhere (14). Another challenge with surgical resection alone is the high rate of recurrence after resection; a study by Sheehan et al. reported a local recurrence rate of 88% for surgery alone (9). In our study, 14 of 28 tumors (50%) were recurrences after complete resection and before CK treatment, and the median time to CK from resection was 2 months (range 1–96). Multiple resections are not an attractive option for such patients, and they present considerable surgical risk and trauma. Stereotactic radiosurgery (SRS) combines the efficacy of resection with a lower rate of radiotherapy-induced morbidity (11), and may be much more suitable for this highly-vascular tumors (15).

Many studies have demonstrated the important role of adjuvant radiotherapy following surgical resection for intracranial HPC. In the most recent systematic review of 523 patients with HPC, adjuvant radiation led to a longer median survival of 123 months in comparison with the 93 months in patients who did not receive it, (p < 0.0001) (13). In another study by Sheehan et al, the local recurrence rate was reported as 88% with surgery alone, which compared with 12.5% with surgery and adjuvant radiotherapy (9). An external beam radiation dose >50 Gy was suggested to give the greatest benefit in previous series (16, 17), but it was not effective in preventing metastasis (7).

Compared with conventional radiotherapy, radiosurgery can achieve a steep dose gradient that minimizes the radiation delivered to surrounding areas (1). Gamma Knife Radiosurgery (GKRS) has become a well-established treatment option for various intracranial tumors and it can administer an accurately-focused high dose of radiation in a single session. In 1993, Coffey et al. published the first preliminary SRS report on HPCs treated using GKS (Elekta Instruments, Tucker, Georgia). The overall tumor control rate was 81.8% after a median follow-up of 14.8 months. Veeravagu et al. summarized 11 published studies on stereotactic radiosurgery covering a total of 137 patients with 241 recurrent and residual HPC tumors treated between 1987 and 2010. They found a mean tumor control rate of 81.3% after a mean follow-up period of 37.2 months, with a mean prescription margin dose of 16.2 Gy (11).

**Table 2** summarizes 16 published studies (including this present series) on the use of stereotactic radiotherapy for recurrent and residual HPCs. Between the years of 1987 and 2019, a total of 294 patients with 529 lesions were treated with stereotactic radiotherapy and were reported in the literature. For these lesions, the mean prescription dose to the tumor margin was 16.7 Gy, the mean follow-up period was 39.9 months, and the mean tumor control rate was 75.2% (1, 2, 5, 6, 9, 10, 12, 14, 17–23). Our series covered 15 patients with 28 tumors, and at a mean margin dose of 21.2 Gy, the control rate was 78.5% after 34.5 months of follow-up, a control rate that is higher than the mean of the previous studies. Higher prescription doses seem to translate to increased tumor control rates. The average total tumor control rates of GKRS series (14) and CK series (3) over the sixteen published studies are 74.7 and 78.4%, respectively. Postoperative SRT seems to provide an effective and safe adjuvant management option for patients with residual, recurrent and metastatic intracranial HPCs. However, determination of the best stereotactic radiotherapy option requires more results from large, multi-center series.

**Table 2:** Literature review of previous studies that reported control rates in patients treated for hemangiopericytoma with SRT.

Authors	Institution	Study period	Number of patients/Lesions	Volume (ml),mean (range)	Type of radiotherapy	Number of fractions	Prescription dose (Gy), mean(range)	Follow-up (months), mean (range)	Tumor control at last follow-up (%)	Intracranial metastasis, n (%)	Extraneural metastasis, n (%)	Extraneural metastasis site
Coffey et al. (9)	Mayo Clinic	1990–1992	5/11	8.53(0.40–24.25)	GK	1	15.5(12–18)	14.8(10–17)	81.8	2(40)	1(20)	Liver, spinal
Galanis et al. (17)	Mayo Clinic	1976–1996	10/20(Includes five patients from Coffey et al.)	–	GK	1	(12–18)	−(6-36)	100	–	–	–
Payne et al. (18)	U of Virginiac	1991–1999	10/12	7.6(0.3–33.6)	GK	1	14(2.8–25)	24.8(3–56)	75	2(20)	0	–
Sheehan et al. (14)	U of Pittsburgh	1987–2001	14/15	8.8(0.3–26.6)	GK	1	15(11–20)	31.3(5–76)	80	2(14.5)	2(14.5)	Spinal, rib, lung
Chang et al. (5)	Stanford	1992–2002	8/8	–	LINAC,CK	1	20.5(16–24)	44(8–77)	75	0	1 (12.5)	Temporalis muscle
Ecker et al (11)	Mayo Clinic	1980–2000	15/45(Includes five patients from Coffey et al)	7.8(0.4–58.3)	GK	1	16 (12–21)	45.6 (–)	93	4(don’t mention the site)(27)	0	
Kano et al. (6)	U of Pittsburgh	1989–2006	20/29	4.5(0.07–34.3)	GK	1	15(10–20)	37.9(–)	72.4	3(15)	3+2 both intracranial and extraneural (25)	Lung, liver, rib, neck, vertebral body
Sun et al. (19)	Beijing Neu. Ins	1994–2006	22/58	5.4(0.4–31.2)	GK	1	13.5(10–20)	26(5–90)	89.7	7(31.8)	3(13.6)	Orbit, bone, liver, lungs, pleura, subcutis
Iwai et al. (20)	Osaka City Hosp	–	5/6	11(4.5–18.8)	GK	1	13.7(12–16)	34(10–48)	66.7	1(20)	1(20)	Lung
Olson et al. (1)	U of Virginia	1989–2008	21/28	4.6(0.3–18.7)	GK	1	17(2.8–22)	69(2–138)	46.4	3(14)	4(19)	Liver, lung, kidney, bone, bowel, external auditory canal
Veeravagu et al. (10)	Stanford	2002–2009	12(Spine 3)/22(Spine 9)	9.16(0.03–56.7)	CK	1-5	21.2(16–30)	37(10–73)	81.8	–	–	–
Tsugawa et al. (21)	Nagoya Kyoritsu Hospital	2004–2010	7/10	5.2(0.3–23.9)	GK	1	17.5(10–20)	52.1 (13–71)	69.7	2(28.5)	1(14.2)	–
Copeland et al. (22)	Mayo Clinic	1990–2010	22/64	3.3 (0.1–58.3)	GK	1	15(12–21)	31(1–155)	59	9(40.9)	–	–
Kim et al. (23)	Kosin University Gospel Hospital	2002–2014	18/40	1.2 (0.4–7.4)	GK	1	20.5(13–30)	71.8 (3.3–153.3)	80	8 (44.4)	7 (38.9)	Liver, spine, lung, kidney, bone, pancreas
Cohen-Inbar (2)	Multicenter	1988–2014	90/133	4.9(0.2–42.4)	GK	1	15(2.8–24)	59 (6–183)	54.8	25(27.8)	22(24.4)	Liver, lung, kidney, bone, bowel, and external auditory canal
Present study	PLA General Hospital	2014–2019	15/28	13.06(0.84–67.2)	CK	1-3	21.1(14–27)	34.5(1–77)	78.5	3(20)	1(6.6)	Liver, spine

SRT, stereotactic radiotherapy; GK, Gamma Knife; CK, CyberKnife.

The CK system is one available device for stereotactic radiotherapy. The system offers both single- and multi-session SRT options. The two previously published studies on patients with HPC treated using CK SRT were both from Stanford. The first series was conducted by Chang and Sakamoto in 2003 (5) and demonstrated tumor control in 75% of the HPCs treated during a mean follow-up time of 44 months. Although the mean radiosurgery dose to the tumor margin was higher (20.5 Gy) than that from GKRS in another series (16.2 Gy), there were no radiosurgery related complications and very ideal tumor control rates. In the second series, the tumor margin dose was slightly higher (21.5 Gy) than in the first series (20.5 Gy). The rates of tumor reduction, stability, recurrence, and total tumor control were 54.5, 27.3, 18.2, and 81.8%, respectively. The tumor margin dose in our series was 21.2 Gy, and the rates of tumor reduction, stability, recurrence, and total tumor control were 75, 3.5, 21.4, and 78.5%, respectively. Although the rate of tumor control was similar to the previous two series, the follow-up time in our study was not long enough. A satisfactory result in our study was the disappearance of seven tumors over the relatively short follow-up time, although the longer-term outcomes require further observation. In the six tumors that recurred after CK therapy, and the marginal doses were 27, 16, 22.5, 19.5, 22.5, and 22.5 Gy, and the grades were III, II–III, III, II, II, and II, respectively. There is no obvious correlation between the marginal dose and the grade. Sun et al. considered that a high margin dose appeared to achieve a reduction in the rate of local recurrence (19). Therefore, a higher prescription dose needs to be tried gradually for HPCs. Moreover, we found that four of 15 patients in the current series whose tumor arose at the deep of the brain had a poor prognosis.

Previous studies reported that the most common sites of extraneural metastases were the lungs, bones, liver, intra-abdominal and subcutaneous tissues, breast, pleura, and thyroid (13). Galanis et al. noted that bones and liver were the most common metastatic sites (82 and 41% of extraneural recurrences, respectively) in their series (17). Incidences of extracranial metastasis of 13, 33, and 64% at 5, 10, and 15 years have been reported, and their occurrence significantly shortens survival (17). The mean time to extraneural metastasis shows substantial variance, ranging from several months to many years (25), even from initial diagnosis (11). One of our patients currently alive with liver and bone metastases 6 years after diagnosis, with the patient having developed. Two intracranial metastases at 2 and 3 years after diagnosis. The rate of intracranial metastases in the 22 patients of Sun’s series was 31.8% (n = 7), whereas the rate of extracranial metastases was 13.6% (n = 3). In our study, the intracranial metastasis rate was 20% (n = 3) and the extracranial metastasis rate 6.6% (n = 1). The variability in these results is related to the unpredictable nature of HPCs. Other CK SRT series made paid little mention to intracranial or extraneural metastases. Because HPCs are highly aggressive, the initial tumor volume can be regenerated, even if it is reduced or has disappeared after previous treatment (10); in our series, six tumors (1, 9, 11, 14, 19 and 22) showed such a changing course. Stereotactic radiotherapy is a focal localized treatment modality, and the possibility of repeatable treatment is an advantage, but it may also be ineffective in preventing metastasis. Hence, CNS HPC requires long-term follow-up and surveillance for recurrence and metastasis. The appropriate interval is uncertain, but 3 or 6 months might be considered reasonable.

Until now, the use of chemotherapy for treating CNS HPCs has been very disappointing, although chemotherapy may be helpful in the specific case of recurrence after radiotherapy (8). However the efficacies of new therapies for extracranial HPCs, including anti-angiogenesis therapies, are currently being evaluated (26), and these treatments may also be useful for intracranial HPCs. In our series, one patient (number 4) accepted anti-angiogenesis therapies (pazopanib hydrochloride) after liver and bone metastases were found. Partial embolization of the liver metastases was also performed at the same time. We found that the metastatic tumors shrank after 6 months. Programmed cell death ligand-1 (PD-L1) is frequently expressed in intracranial SFT/HPCs, and diffuse or intense PD-L1 expression might be associated with the early occurrence of extracranial metastasis (27). A combination of SRS and targeted drugs may improve the local control rate and extend survival time, but multicenter prospective large-sample clinical trials are needed to confirm this.

The main limitations of our study are the retrospective design, the small number of patients, and the relatively short follow-up. Although no severe complications occurred after a mean follow-up period of 34.5 months, the follow-up periods were insufficient. We also failed to identify the relationship between dose and volume change. Obviously, much longer, follow-up and a larger population will be required to establish the long-term efficacy of CK-based SRT for HPC. Therefore, we must be cautious with our conclusions, because these findings are preliminary. Although a longer follow-up period and a larger population are necessary to confirm these early results, this analysis documenting our preliminary experience of CK-based SRT for intracranial HPCs is very encouraging.

## Conclusions

On the basis of the current results, fractioned radiotherapy using CyberKnife is an effective and safe option for the management of intracranial HPCs after surgical resection. Our patients experienced total tumor control rates similar to those described in previous studies. Therefore, SRT using CK can be considered an important adjuvant radiation treatment for residual, recurrent, and metastatic intracranial HPCs.

## Data Availability Statement

The original contributions presented in the study are included in the article/**Supplementary Material**. Further inquiries can be directed to the corresponding authors.

## Ethics Statement

The studies involving human participants were reviewed and approved by Institutional Review Board in PLA General Hospital. The patients/participants provided their written informed consent to participate in this study. Written informed consent was obtained from the individual(s) for the publication of any potentially identifiable images or data included in this article.

## Author Contributions

LH prepared for the writing of manuscript preparation. YZ performed the data analyses. JB prepared for MRI data analysis. ZC performed literature research. ZZ performed clinical studies. JL performed statistics of treatment plan parameters. JW performed statistical analysis. XY proposed amendments to the manuscript. ZL corrected the manuscript. BQ helped perform the analysis with constructive discussions. LP designed the experiment. All authors contributed to the article and approved the submitted version.

## Conflict of Interest

The authors declare that the research was conducted in the absence of any commercial or financial relationships that could be construed as a potential conflict of interest.

## Abbreviations

HPCs, hemangiopericytomas; CK, CyberKnife; GK, Gamma Knife; MRI, Magnetic Resonance Imaging; SRT, Stereotactic Radiotherapy; GKRS, Gamma Knife Radiosurgery.
