# Supplementary material for: Treatment of Residual, Recurrent, or Metastatic Intracranial Hemangiopericytomas With Stereotactic Radiotherapy Using CyberKnife
Source: Front Oncol. 2021 Mar 3;11:577054. doi: 10.3389/fonc.2021.577054 (PMC7982841; doi:10.3389/fonc.2021.577054)
Supplement: Supplementary file 1 [file Table_1.docx]

| TABLE 1 **\|** Tumor characteristics and radiosurgical parameters | | | | | | | | | | | | | | | | |
| --- | --- | --- | --- | --- | --- | --- | --- | --- | --- | --- | --- | --- | --- | --- | --- | --- |
| **PtNo.** | **LnNo.** | **Age at CK** | **Site** | **Residual or recurrent** | **Tumor volume (cc)** | **Tumor volume (cc)fellow up** | **Marginal dose (Gy)** | **Isodose Line (%)** | **Fractions** | **Dmax (Gy)** | **Mean(Gy)** | **Dmin(Gy)** | **Conformality index** | **Fellow up time** | **Tumor Control at last F/U** | **Metastases** |
| 1 | 1 | 58 | Left temporal lobe | RC | 2.09 | 8.79 | 27 | 80 | 3 | 33.75 | 30.57 | 26.52 | 1.13 | 35 | RC after RD | Intracranial metastases |
|  | 2 | 60 | Left anterior skull base | MT | 2.02 | 0 | 27 | 80 | 3 | 33.75 | 30.83 | 15.47 | 1.2 | 47 | DS |  |
|  | 3 | 61 | Left temporal lobe | RC | 8.79 | 0 | 22.5 | 75 | 3 | 30 | 27.05 | 11.96 | 1.17 | 41 | DS |  |
|  | 4 | 61 | The fourth ventricle | MT | 0.84 | 0 | 14 | 70 | 1 | 20 | 17.08 | 11.54 | 1.17 | 37 | DS |  |
| 2 | 5 | 49 | Left parietal parasagittal | RS | 5.48 | 0 | 24 | 80 | 3 | 30 | 26.94 | 22.42 | 1.12 | 48 | DS | N |
| 3 | 6 | 38 | Left tentorium cerebelli confluence of sinuses | RC | 5.13 | 0.13 | 24 | 80 | 3 | 30 | 26.7 | 19.14 | 1.19 | 48 | RD | Intracranial metastases |
|  | 7 | 40 | Right tentorium cerebelli confluence of sinuses | MT | 3.78 | 67.2 | 16 | 70 | 1 | 22.85 | 19.44 | 11.84 | 1.19 | 39 | RC after RD |  |
|  | 8 | 43 | Right tentorium cerebelli | RC | 67.2 | 10.2 | 18 | 70 | 3 | 25.71 | 22.49 | 15.48 | 1.11 | 15 | RD |  |
|  | 9 | 43 | Tentorium cerebelli | MT | 0.91 | 0.12 | 20 | 70 | 1 | 28.57 | 24.55 | 19.63 | 1.16 | 15 | RD |  |
| 4 | 10 | 34 | Tentorium cerebelli | RS | 38.5 | 4.5 | 21 | 80 | 3 | 26.25 | 23.89 | 15.03 | 1.23 | 77 | RD | Liver,Thoracic11，Lumbar4 |
|  | 11 | 36 | Tentorium cerebelli | RC | 20.86 | 7.85 | 22.5 | 70 | 3 | 32.14 | 27.66 | 4.02 | 1.18 | 17 | RC after RD |  |
|  | 12 | 37 | Tentorium cerebelli | RC | 7.85 | 0.4 | 18 | 70 | 3 | 25.71 | 22.79 | 12.22 | 1.17 | 38 | RD |  |
|  | 13 | 39 | Tentorium cerebelli | RC | 7.2 | 0.5 | 18 | 70 | 3 | 25.71 | 21.87 | 7.2 | 1.33 | 19 | RD |  |
| 5 | 14 | 32 | Right cavernous sinus | RS | 4.03 | 10.5 | 19.5 | 70 | 3 | 27.85 | 24.57 | 14.83 | 1.19 | 55 | RC after RD | N |
|  | 15 | 34 | Right orbital apex | RC | 5.17 | 0 | 21 | 75 | 3 | 28 | 25.35 | 6.58 | 1.2 | 42 | DS |  |
| 6 | 16 | 37 | Sinus-related anterior skull base | RS | 8.61 | 2.52 | 22.5 | 80 | 3 | 28.12 | 25.23 | 17.17 | 1.29 | 58 | RD | N |
|  | 17 | 41 | Sinus-related anterior skull base | RC | 1.25 | 0.6 | 22 | 70 | 2 | 31.42 | 28.2 | 20.49 | 1.4 | 23 | RD |  |
| 7 | 18 | 43 | Right cerebellum | RS | 11.33 | 0 | 21 | 75 | 3 | 28 | 24.93 | 16.07 | 1.22 | 39 | DS | N |
| 8 | 19 | 49 | Right parietal parasagittal | RC | 3.61 | 6.42 | 22.5 | 70 | 3 | 32.14 | 28.29 | 20.14 | 1.36 | 25 | RC after RD | N |
| 9 | 20 | 48 | Right tentorium cerebelli | RC | 13.41 | 7.04 | 21 | 70 | 3 | 30 | 26.94 | 17.16 | 1.4 | 13 | RD | Leptomeningeal metastasis |
| 10 | 21 | 46 | Parietal lobe | RS | 11.81 | 0 | 21 | 70 | 3 | 30 | 25.75 | 15.4 | 1.19 | 49 | DS | N |
| 11 | 22 | 34 | Confluence of sinuses | RS | 21.47 | 34.76 | 21 | 80 | 3 | 26.2 | 23.98 | 16.7 | 1.12 | 36 | RC after RD | N |
|  | 23 | 37 | Confluence of sinuses | RC | 34.76 | 1.92 | 22.5 | 70 | 3 | 32.14 | 28.29 | 18.39 | 1.11 | 34 | RD |  |
| 12 | 24 | 42 | Right parietal | RS | 46.84 | 16.12 | 18 | 70 | 3 | 25.71 | 23.18 | 10.37 | 1.31 | 31 | RD | N |
| 13 | 25 | 46 | Left cerebellum | RS | 15.61 | 3.12 | 21 | 70 | 3 | 30 | 25.61 | 18.62 | 1.23 | 24 | RD | N |
|  | 26 | 46 | Left cerebellum | RS | 8.07 | 1.62 | 21 | 70 | 3 | 30 | 24.99 | 18.51 | 1.23 | 24 | RD |  |
| 14 | 27 | 42 | Sellar region | RS | 4.35 | 3.55 | 22.5 | 70 | 3 | 32.1 | 29.09 | 18.33 | 1.26 | 19 | ST | N |
| 15 | 28 | 46 | Right parietal | RS | 4.84 | 0.5 | 22.5 | 70 | 3 | 32.14 | 28.68 | 19.57 | 1.45 | 19 | RD | N |

PtNo, patient number; LnNo, lesion number; F/U, follow-up; RC, recurrent; RS, residual; MT, metastatic; RD, reduction; ST, stable; DS, disappeared; N, none.
