# Supplementary material for: Treatment of Residual, Recurrent, or Metastatic Intracranial Hemangiopericytomas With Stereotactic Radiotherapy Using CyberKnife
Source: Front Oncol. 2021 Mar 3;11:577054. doi: 10.3389/fonc.2021.577054 (PMC7982841; doi:10.3389/fonc.2021.577054)
Supplement: Supplementary file 2 [file Table_2.docx]

|  |  |
| --- | --- |
| TABLE 2 **\|** Summary of patients characteristics and radiosurgical parameters. | |
| Number of Patients | 15 |
| Sex |  |
| Male | 8 (53.4%) |
| Female | 7 (46.6%) |
| Age (years) Mean, Median (range) | 43.2, 43, (32-58) |
| Number of Tumors | 28 |
| Residual (Pre-CK) | 10 (35.7%) |
| Recurrent (Pre-CK) | 14 (50.0%) |
| Metastatic | 4 (14.3) |
| FU duration after CK (months) Mean, Median (range) | 34.5 , 35.5（1-77） |
| Tumor volume (cm^3^) Mean, Median (range) | 13.06，6.34（0.84-67.2） |
| Tumor volume fellow up (cm^3^) Mean, Median (range) | 6.72，1.11 (0-67.2) |
| Marginal dose (Gy) Mean, Median (range) | 21.1，21（14-27） |
| Isodose Line Mean, Median (range) | 73.03%, 70.0% (70% - 80%) |
| Maximum Dose (Gy) Mean, Median (range) | 28.80，29.28（20-33.75） |
| Target volume treated at/above dose Mean, Median (range) | 96.42%, 96.39%（90%-99.26%） |
| Time to CK Post Surgery（month）Mean,Median (range) | 14.5, 2 (1 - 96) |
| Tumor Disappeared | 7 (25%) |
| Tumor Reduction | 14 (50%) |
| Tumor Stable | 1 (3.07%) |
| Tumor Recurrence after Reduction | 6 (21.4%) |
| Total Tumor Control | 22 (78.5%) |
| Patients intracranial metastasis | 3 (20.0%) |
| Patients extraneural metastasis | 1 (6.6%) |
